# Supplementary material for: Nelfinavir Induces Cytotoxicity towards High-Grade Serous Ovarian Cancer Cells, Involving Induction of the Unfolded Protein Response, Modulation of Protein Synthesis, DNA Damage, Lysosomal Impairment, and Potentiation of Toxicity Caused by Proteasome Inhibition
Source: Cancers (Basel). 2021 Dec 26;14(1):99. doi: 10.3390/cancers14010099 (PMC8750028; doi:10.3390/cancers14010099)
Supplement: Supplementary file 1 [file cancers-14-00099-s001.zip › cancers-1530866-supplementary.pdf]

Table S1. Source and dilutions of antibodies utilized in this work.

**Antibody**	**Clone or catalogue**	**Company**	**Concentration**
PERK	3192S	Cell Signaling Technology	1:1000
GRP78	3177S	Cell Signaling Technology	1:1000
IRE1α	3294S	Cell Signaling Technology	1:1000
p-eIF1α	3398S	Cell Signaling Technology	1:1000
eIF2α	9722S	Cell Signaling Technology	1:1000
ATF4	11815S	Cell Signaling Technology	1:1000
CHOP	5554S	Cell Signaling Technology	1:1000
ATF6	NBP1-40256	Novus Biologicals	1:1000
Puromycin	MABE343	EMD Millipore Corporation	1:20000
p-Akt (Ser473)	4058L	Cell Signaling Technology	1:1000
p-Akt (Thr308)	4056L	Cell Signaling Technology	1:1000
Akt	2920S	Cell Signaling Technology	1:1000
p-ERK	9106L	Cell Signaling Technology	1:1000
ERK	9102L	Cell signaling Technology	1:1000
p27	610242	BD Biosciences	1:1000
γH2AX	05-636	EMD Millipore Corporation	1:1000
p-KAP1 (Ser824)	NB100-2350	Novus Biologicals	1:1000
LC3B	3868S	Cell Signaling Technology	1:1000
Cdk2	sc-6248	Santa Cruz Biotechnology	1:500
p-Cdk2 (Thr160)	2561S	Cell Signaling Technology	1:1000
Ubiquitin	3933S	Cell Signaling Technology	1:1000
β-Actin	A5441	Sigma Life Sciences	1:10000
Caspase-7	12827S	Cell Signaling Technology	1:1000
Bax	5023S	Cell Signaling Technology	1:1000
Bcl-2	15071S	Cell Signaling Technology	1:1000
Anti-mouse	170-6516	BioRad Laboratories Inc.	1:8000
Anti-rabbit	170-6515	BioRad Laboratories Inc.	1:10000

Table S2. Concentration of nelfinavir (NFV) needed to achieve 50% reduction in clonogenic survival (IC_{50}) of the HGSOC cell lines studied.

**Cell Line**	**IC_{50} (μM)**
PEO1	10.05 ± 0.75
PEO4	12.40 ± 2.60
PEO6	11.40 ± 0.40
PEO14	16.07 ± 4.90
PEO23	11.14 ± 0.71

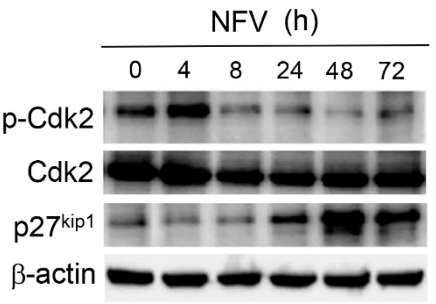

Figure S1. Inhibition of Thr160 phosphorylation of Cdk2 (p-Cdk2) by nelfinavir (NFV) correlates with increased levels of Cdk inhibitor p27^{kip1}. PEO1 cells were incubated with 20 µM NFV for the depicted times. At the end of the experiment, total protein extracts were obtained, and western blotting assessed the expression of p-Cdk2, Cdk2, p27^{kip1}, and β-actin.

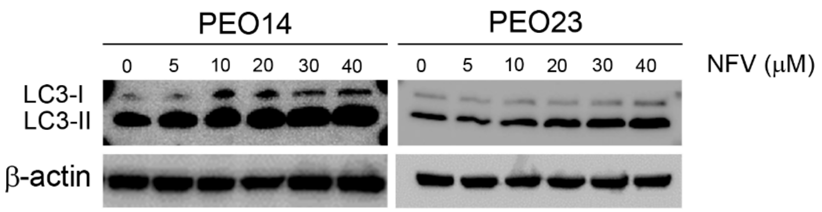

Figure S2. In PEO14 and PEO23 cells treated with various concentrations of nelfinavir (NFV) for 72 h; the autophagosome-related protein LC3II increases in response to NFV in a concentration-dependent manner.

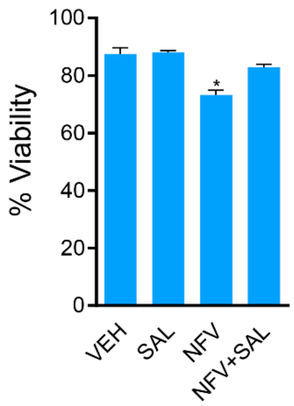

Figure S3. The cytotoxicity of nelfinavir (NFV) towards PEO1 ovarian cancer cells is significantly prevented by the small molecule salubrinal. PEO1 cells were treated vehicle (VEH) or with 20 µM NFV, with or without concurrent 50 µM salubrinal (SAL) for 72 h. At the end of the experiment, the cells were trypsinized and subjected to microcytometry analysis to attain their viability. *$p<0.05$ compared to NFV (One-way ANOVA followed by Tukey's Multiple Comparison Test).

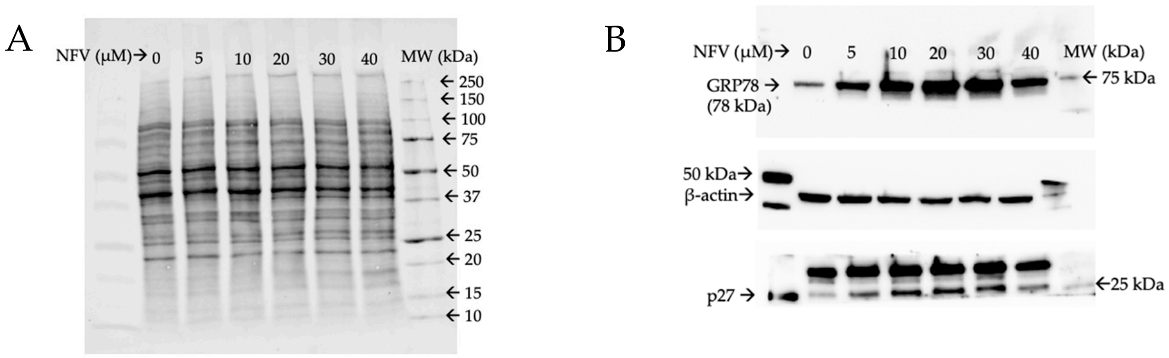

Blot 1. Proteins extracted from PEO1 cells treated with 5-40 μM NFV were run for 30 min at 200V on TGX stain free fast cast acrylamide gel (12%). After the run, the gel was activated by UV light. Proteins on the gel were transferred using TransBlot Turbo for 7 min. After transfer, the total protein load was visualized in the unstained membrane using BioRad ChemiDoc imager (A). After 1 h of blocking with 5% non-fat dry milk, blot 1 was cut between 37 and 25 kDa, and between 25 and 20 kDa. The top part above 37 kDa was incubated for GRP78 and the bottom part above 25 kDa for p27^{kip1} (B). After that the area between 50 kDa and 37 kDa was cut and incubated for β-actin (B). Two stained molecular weights (MW) on each side were used for cutting accuracy. Data from blot 1 were presented in **Figure 2A**.

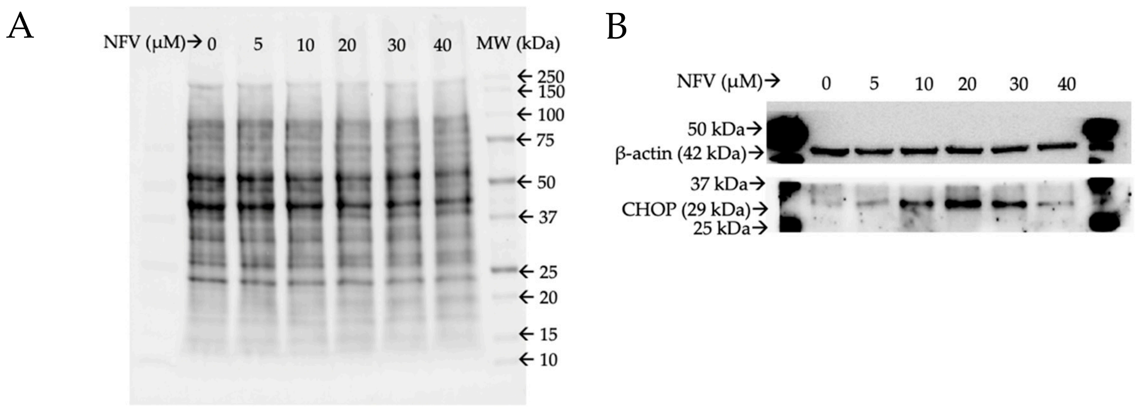

Blot 2. Proteins extracted from PEO1 cells treated with 5-40 μM NFV were run for 30 min at 200V on TGX stain free fast cast acrylamide gel (12%). After the run, the gel was activated by UV light. Proteins on the gel were transferred using TransBlot Turbo for 7 minutes. After transfer, the total protein load was visualized in the unstained membrane using BioRad ChemiDoc imager (A). After 1 h of blocking with 5% non-fat dry milk, blot 2 was cut below 75 kDa, at 37 kDa and at 25 kDa. The part below 75 kDa and above 37 kDa was incubated for β-actin, the part between 37 kDa to 25 kDa was incubated for CHOP. Two stained molecular weights (MW) on each side were used for cutting accuracy. Data from blot 2 were presented in **Figure 2A**.

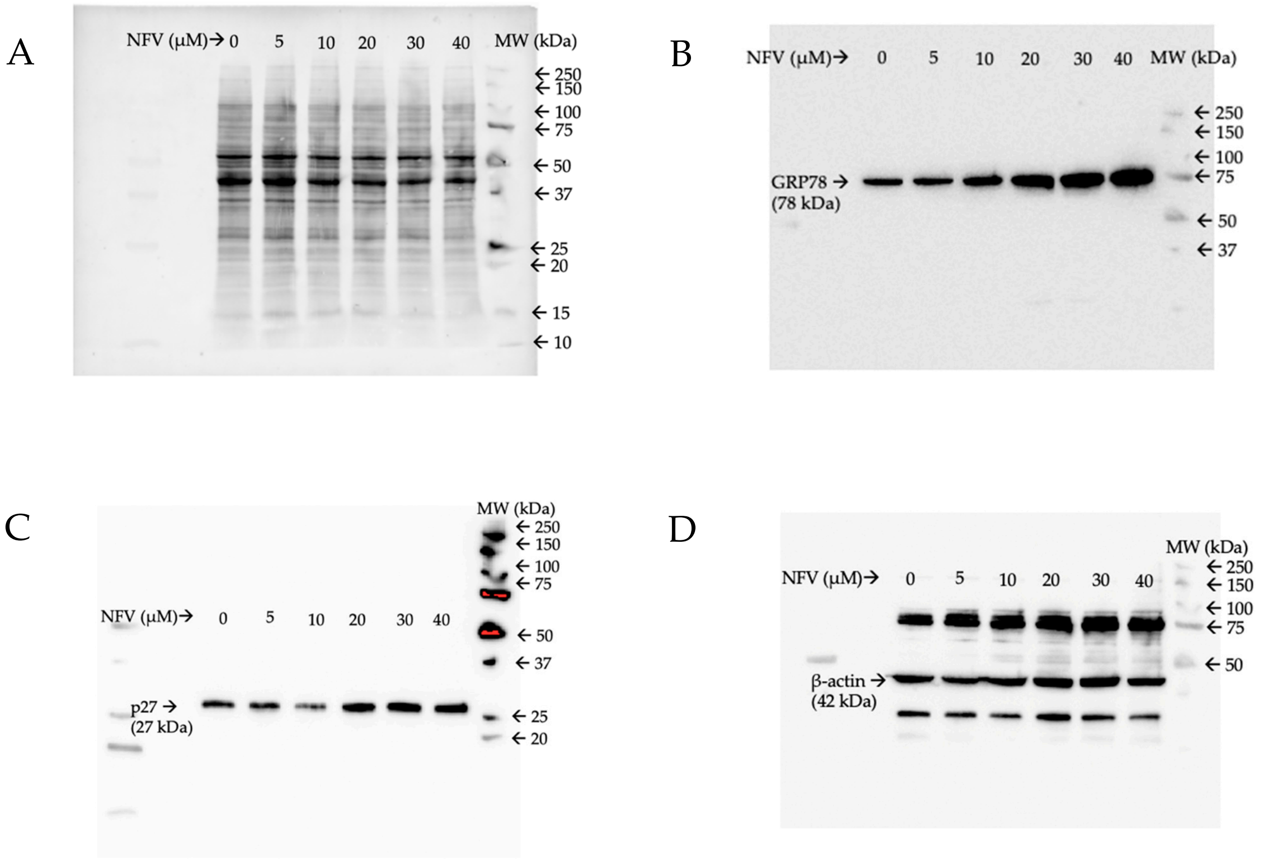

Blot 3. Proteins extracted from PEO14 cells treated with 5-40 µM NFV were run for 30 min at 200V on TGX stain free fast cast acrylamide gel (12%). After the run, the gel was activated by UV light. Proteins on the gel were transferred using TransBlot Turbo for 7 min. After transfer, the total protein load was visualized in the unstained membrane using BioRad ChemiDoc imager (A). After 1 h of blocking with 5% non-fat dry milk, blot 3 was sequentially incubated for GRP78 (B), p27^{kip1} (C), and β-actin (D). Data from blot 3 were presented in **Figure 2A**. MW=molecular weight.

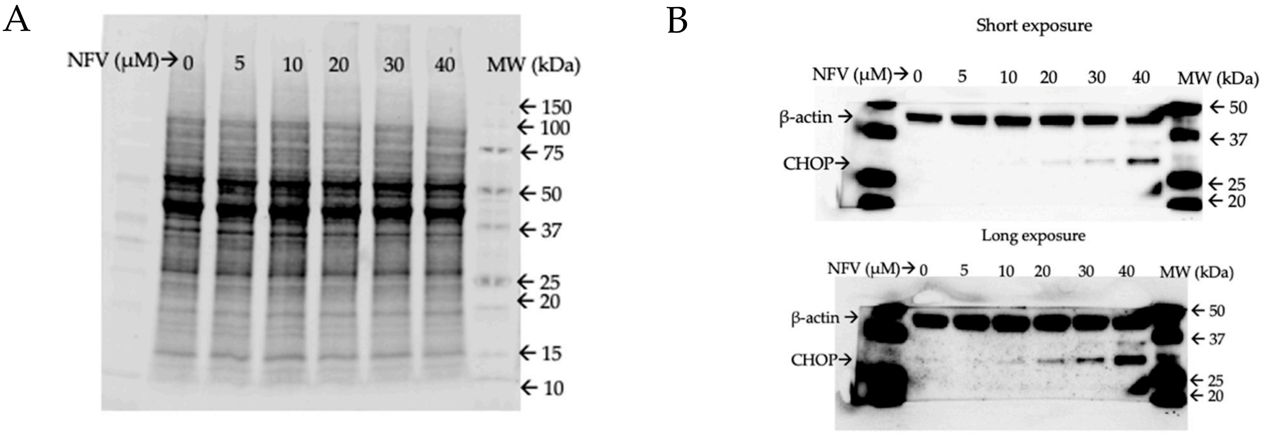

Blot 4. Proteins extracted from PEO14 cells treated with 5-40 µM NFV were run for 30 min at 200V on TGX stain free fast cast acrylamide gel (12%). After the run, the gel was activated by UV light. Proteins on the gel were transferred using TransBlot Turbo for 7 min. After transfer, the total protein load was visualized in the unstained membrane using BioRad ChemiDoc imager (A). After 1 h of blocking with 5% non-fat dry milk, blot 4 was cut below 75kDa and below 20kDa, and the middle part was sequentially incubated for β-actin and CHOP (B). Two stained molecular weights (MW) on each side were used for cutting accuracy. Data from blot 4 were presented in **Figure 2A**.

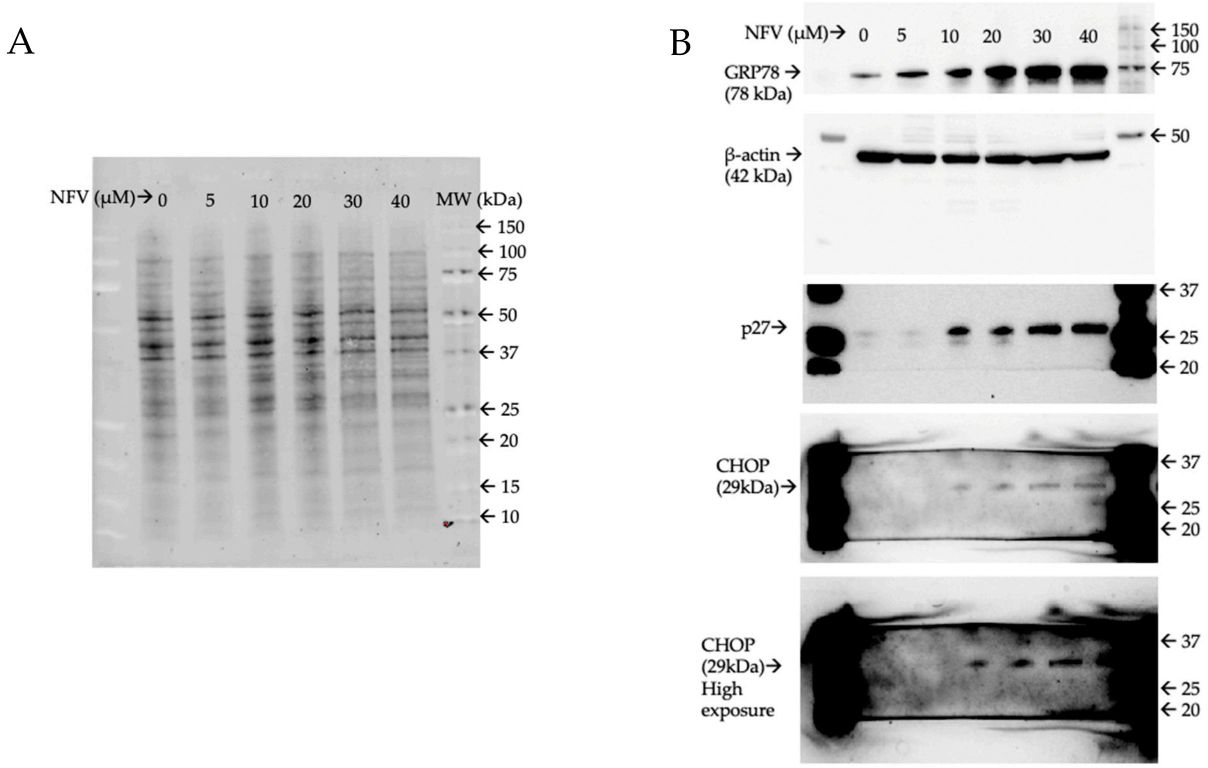

Blot 5. Proteins extracted from PEO4 cells treated with 5-40 µM NFV were run for 30 min at 200V on TGX stain free fast cast acrylamide gel (12%). After the run, the gel was activated by UV light. Proteins on the gel were transferred using TransBlot Turbo for 7 min. After transfer, the total protein load was visualized in the unstained membrane using BioRad chemiDoc imager (A). After 1 h of blocking with 5% non-fat dry milk, blot 5 was cut below 75 kDa and the top part was incubated for GRP78 and the bottom part was incubated for β-actin (B). After that, the bottom blot was cut at the level of 37 kDa and the bottom part was sequentially incubated for p27^{kip1} and CHOP (B). Two stained molecular weights (MW) on each side were used for cutting accuracy. Data from blot 5 were presented in **Figure 2A**.

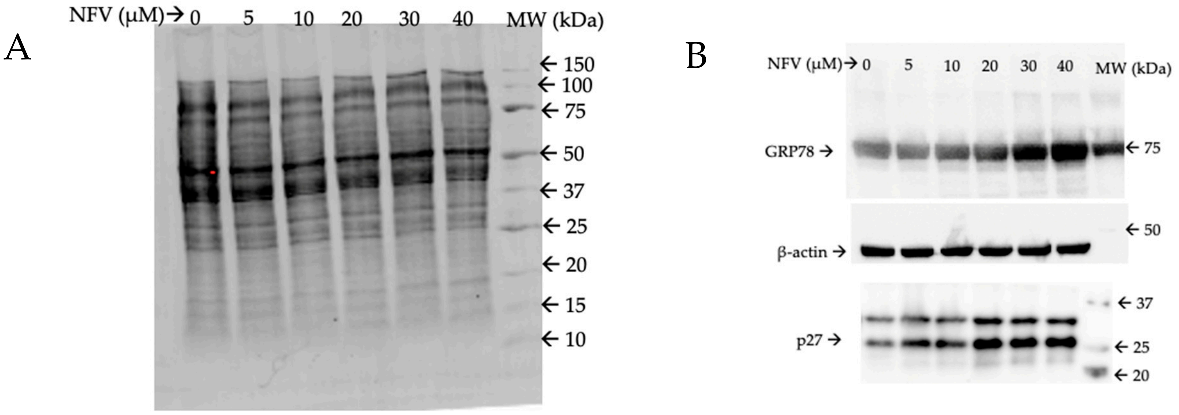

Blot 6. Proteins extracted from PEO23 cells treated with 5-40 µM NFV were run for 30 min at 200V on TGX stain free fast cast acrylamide gel (12%). After the run, the gel was activated by UV light. Proteins on the gel were transferred using TransBlot Turbo for 7 min. After transfer, the total protein load was visualized in the unstained membrane using BioRad ChemiDoc imager (A). After 1 h of blocking with 5% non-fat dry milk blot 6 was cut between 75 and 50 kDa, at 37 kDa and below 20 kDa. The part of the membrane above 75 kDa was incubated for GRP78, the part between 50 and 37 kDa was incubated for β-actin and the part below 37 kDa was incubated for p27^{kip1} (B). Data from blot 6 were presented in **Figure 2A**. MW=molecular weight.

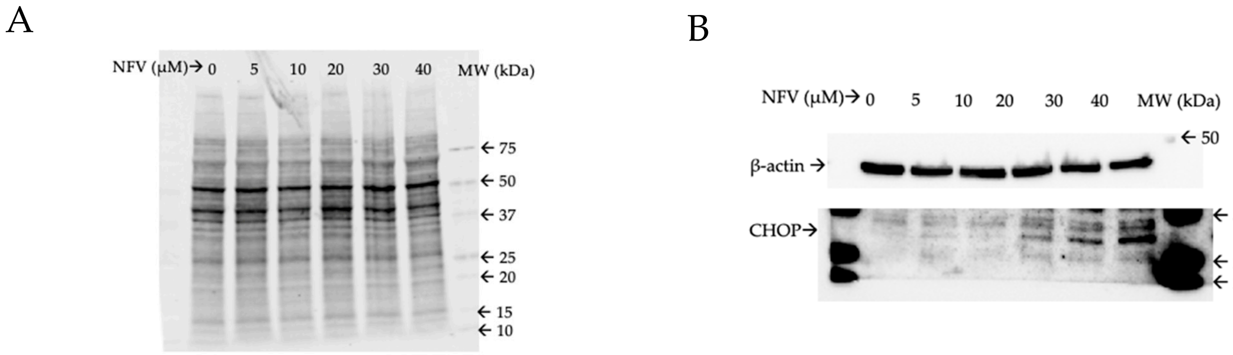

Blot 7. Proteins extracted from PEO23 cells treated with 5-40 µM NFV were run for 30 min at 200V on TGX stain free fast cast acrylamide gel (12%). After the run, the gel was activated by UV light. Proteins on the gel were transferred using TransBlot Turbo for 7 min. After transfer, the total protein load was visualized in the unstained membrane using BioRad ChemiDoc imager (A). After 1 h of blocking with 5% non-fat dry milk, blot 7 was cut at 50 kDa and at 37 kDa and at 20 kDa. Two stained molecular weights (MW) on each side were used for cutting accuracy. The area between 50 and 37 kDa was incubated for β-actin and the area between 37 kDa to 20 kDa was incubated for CHOP (B). Data from blot 7 were presented in **Figure 2A**.

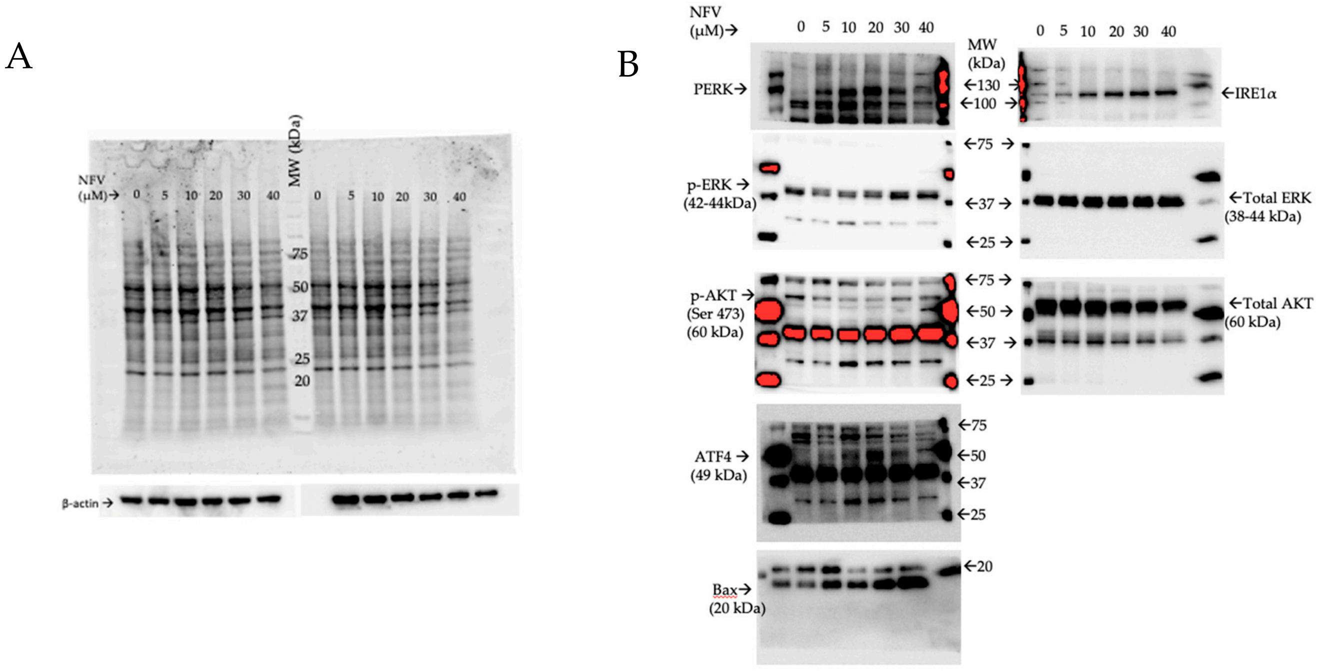

Blot 8. Proteins extracted from PEO1 cells treated with 5-40 µM NFV were run for 30 min at 200V on TGX stain free fast cast acrylamide gel (12%) in two identical sets. After the run, the gel was activated by UV light. Proteins on the gel were transferred using TransBlot Turbo for 7 min. After transfer total protein load was visualized in the unstained membrane using BioRad ChemiDoc imager (A). After 1 h of blocking with 5% non-fat dry milk, the blot was cut in the middle along the molecular weight ladder to yield two identical sets of blots. The blots were cut at 75 kDa and below 25 kDa. The top part was incubated for IRE1α and PERK (B). The middle blots were incubated for p-AKT (Ser 473)/AKT, p-ERK/ERK (B) and β-actin (A). The left middle blot was incubated for ATF4 and the bottommost part was incubated for Bax (B). Two stained molecular weights (MW) on each side were used for cutting accuracy. Data from blot 8 were presented in **Figure 2B, 3B and 5A.**

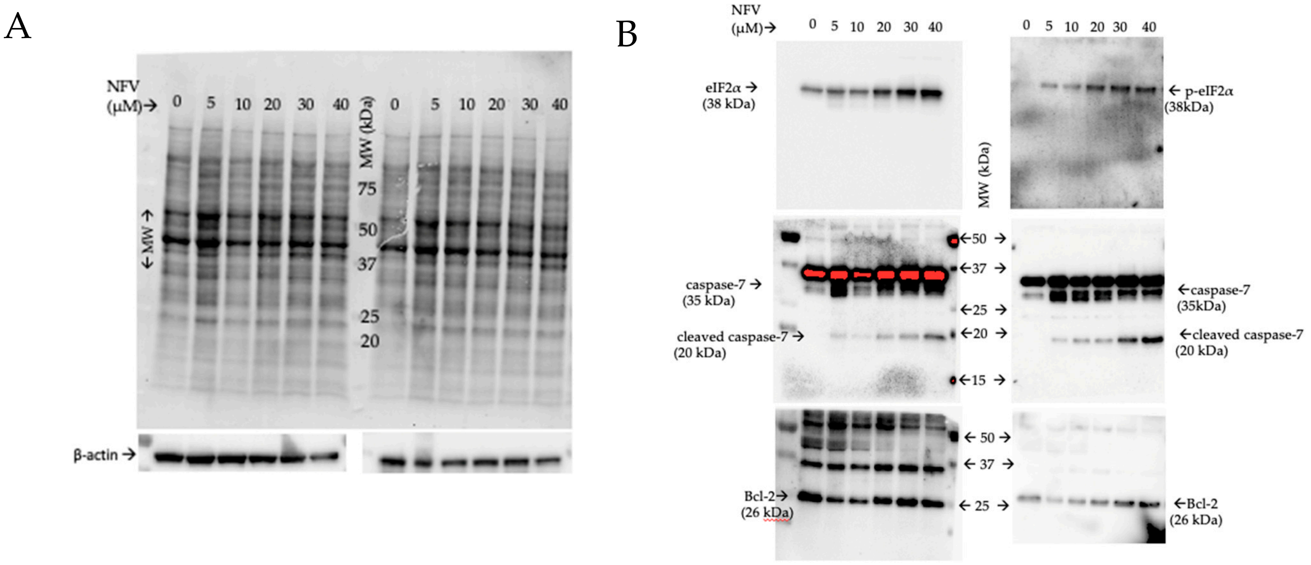

Blot 9. Proteins extracted from PEO1 cells treated with 5-40 μM NFV were run for 30 min at 200V on TGX stain free fast cast acrylamide gel (12%) in two identical sets. After the run, the gel was activated by UV light. Proteins on the gel were transferred using TransBlot Turbo for 7 min. After transfer total protein load was visualized in the unstained membrane using BioRad ChemiDoc imager (A). After 1 h blocking with 5% non-fat dry milk, the blot was cut in the middle along the molecular weight ladder to yield two identical sets of blots. The blots were cut below 75 kDa. The bottom part was incubated for p-eIF2α/eIF2α (B) , caspase-7 (B), Bcl-2 (B) and β-actin (A). Multiple stained molecular weights (MW) were used for cutting accuracy. Data from blot 9 were presented in **Figure 2B and 3B.**

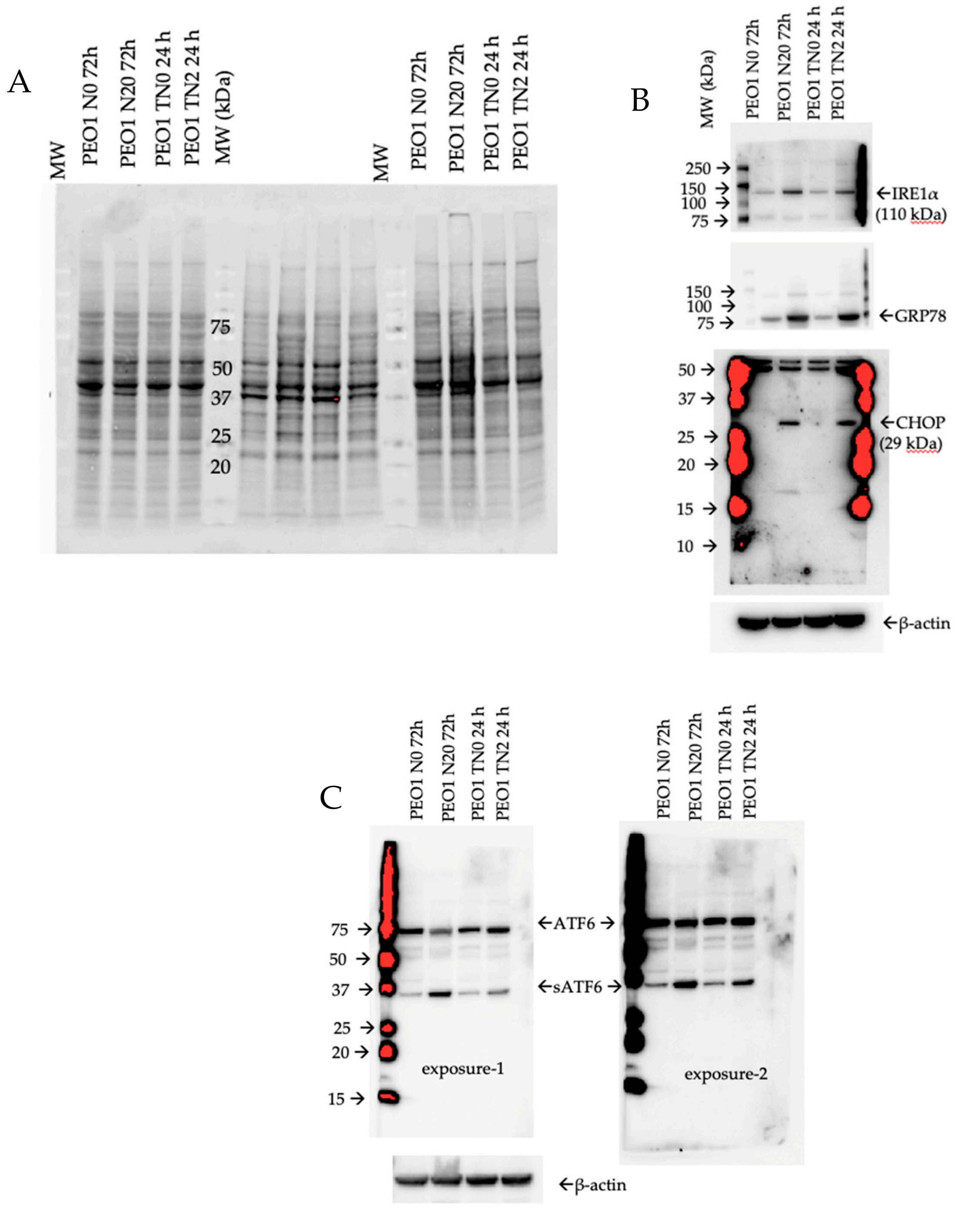

Blot 10. Proteins extracted from PEO1 cells treated with 0 or 20 μM NFV for 72 hours, and 0 or 2 μg/ml tunicamycin (TN) for 24 h were run for 30 min at 200V on TGX stain free fast cast acrylamide gel (12%) in two identical sets. After the run, the gel was activated by UV light. Proteins on the gel were transferred using TransBlot Turbo for 7 min. After transfer total protein load was visualized in the unstained membrane using BioRad ChemiDoc imager (A). After 1 h of blocking with 5% non-fat dry milk, blot 11 was cut in the middle along the molecular weight ladder to yield two identical sets of blots. Left blots was again cut below the mark of 75 kDa. The top part was incubated for IRE1α and GRP78 (B), and the bottom part was incubated for CHOP (B). The right blot was incubated for ATF6 (C). Finally, both blots were incubated for β-actin (B, C). Multiple stained molecular weights (MW) were used for cutting accuracy. Data from blot 10 were presented in **Figure 2C.**

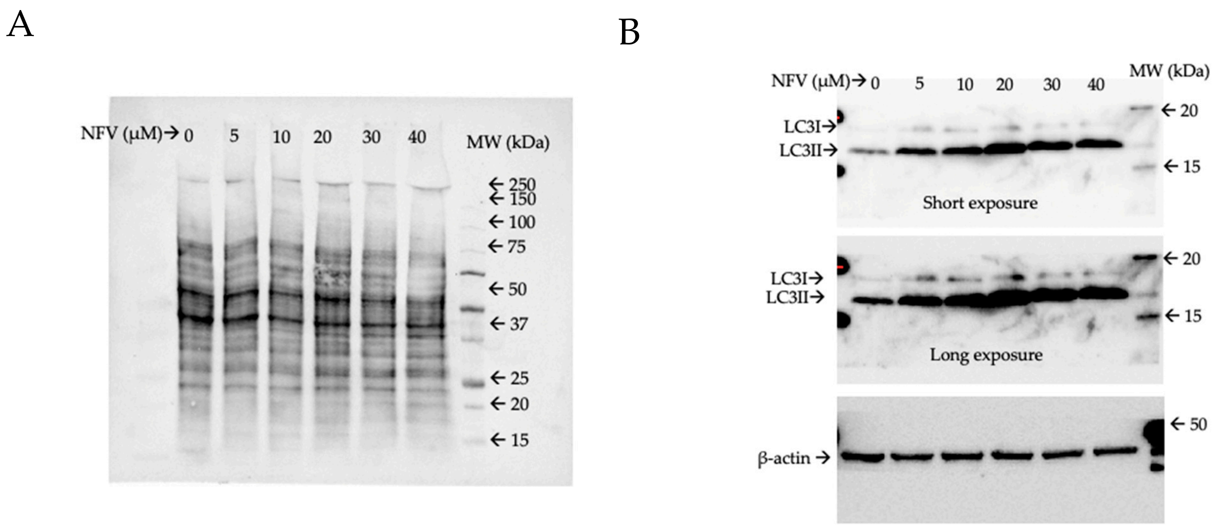

Blot 11. Proteins extracted from PEO1 cells treated with 5-40 μM NFV were run for 30 min at 200V on TGX stain free fast cast acrylamide gel (12%). After the run, the gel was activated by UV light. Proteins on the gel were transferred using TransBlot Turbo for 7 min. After transfer total protein load was visualized in the unstained membrane using BioRad ChemiDoc imager (A). After 1 h of blocking with 5% non-fat dry milk, blot 1 was cut below 75 kDa and below 25Kda. The area below 75 to 25 kDa was incubated for β-actin and the area below 20 kDa was incubated for LC3 (B). Two stained molecular weights on each side were used for cutting accuracy. Data from blot 11 were presented in **Figure 2D.**

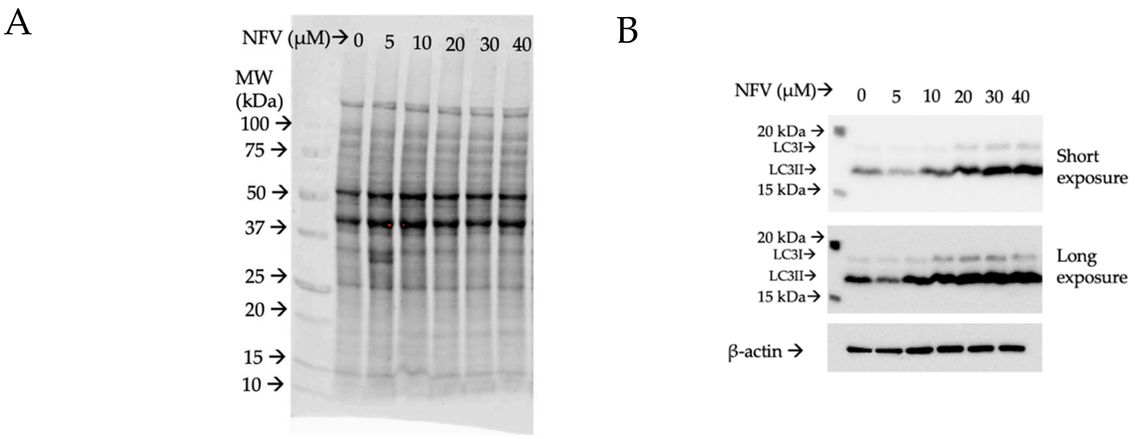

Blot 12. Proteins extracted from PEO4 cells treated with 5-40 μM NFV were run for 30 min at 200V on TGX stain free fast cast acrylamide gel (12%). After the run, the gel was activated by UV light. Proteins on the gel were transferred using TransBlot Turbo for 7 min. After transfer, total protein load was visualized in the unstained membrane using BioRad ChemiDoc imager (A). After 1 h of blocking with 5% non-fat dry milk, blot 1 was cut below 75 kDa and below 25Kda. The area below 75 to 25 kDa was incubated for β-actin and the area below 20 kDa was incubated for LC3 (B). Two stained molecular weights on each side were used for cutting accuracy. Data from blot 12 were presented in **Figure 2D.**

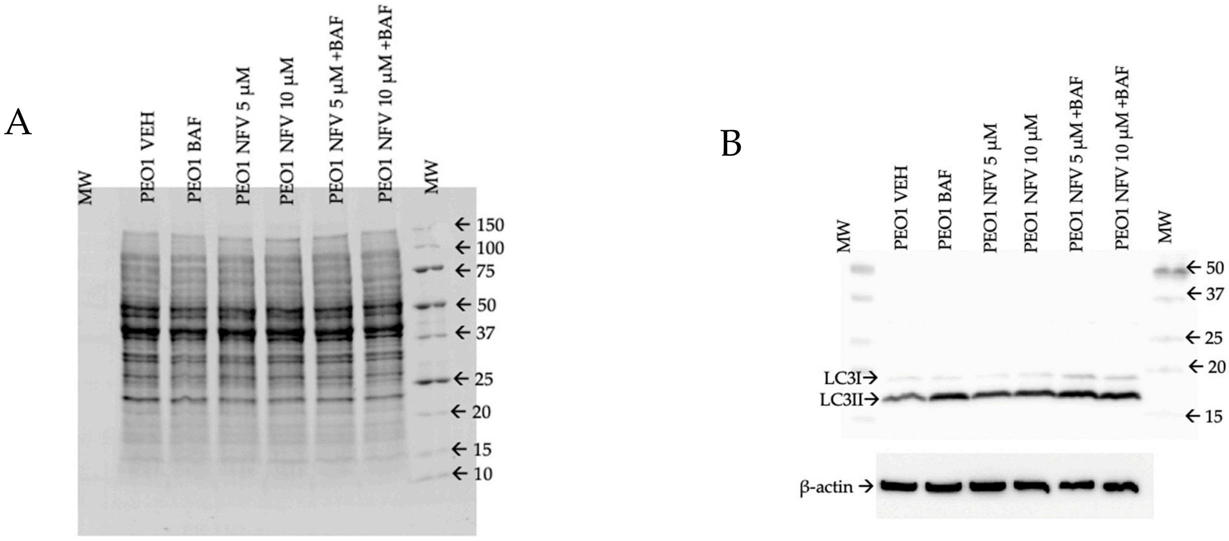

Blot 13. PEO1 cells were treated with 5 or 10 µM of NFV for 72 h. One h before terminating the experiment, the cells were incubated with or without 100 nM of bafilomycin A1 (BAF). Extracted proteins from the samples were run for 30 min at 200V on TGX stain free fast cast acrylamide gel (12%). After the run, the gel was activated by UV light. Proteins on the gel were transferred using TransBlot Turbo for 7 min. After transfer, total protein load was visualized in the unstained membrane using BioRad ChemiDoc imager (A). After 1 h blocking with 5% non-fat dry milk, blot 12 was cut above 50 kDa and the bottom art was incubated for LC3 (B). After that the area between 50 and 37 kDa was cut and incubated for β-actin (B). Two stained molecular weights (MW) on each side were used for cutting accuracy. Data from blot 13 were presented in **Figure 2E.**

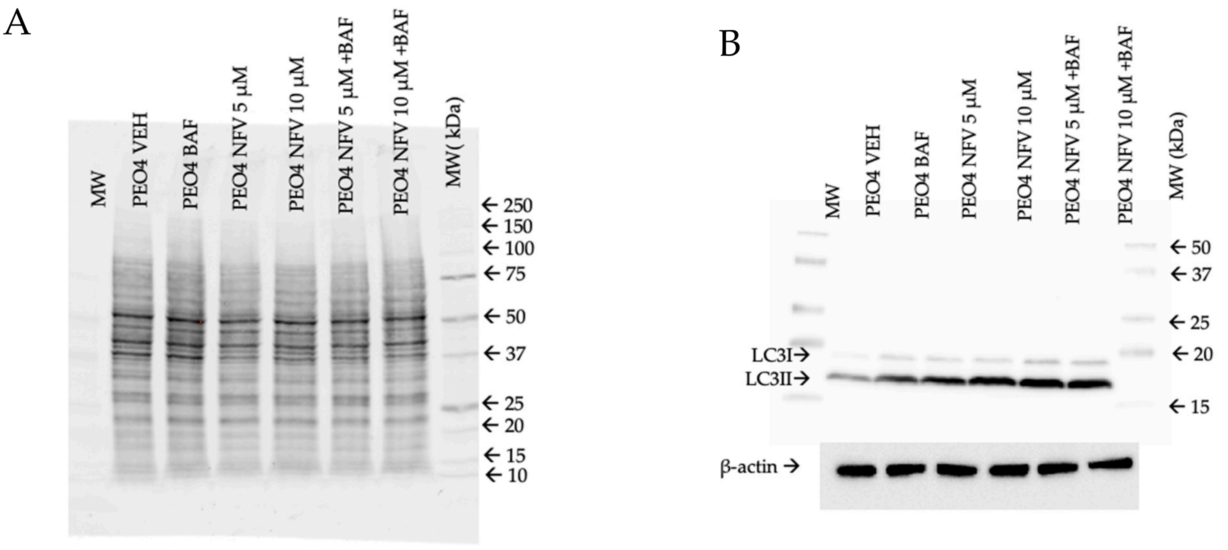

Blot 14. PEO4 cells were treated with 5 or 10 µM of NFV for 72 h. One h before terminating the experiment, the cells were incubated with or without 100 nM of bafilomycin A1 (BAF). Extracted proteins from the samples were run for 30 min at 200V on TGX stain free fast cast acrylamide gel (12%). After the run, the gel was activated by UV light. Proteins on the gel were transferred using TransBlot Turbo for 7 min. After transfer, total protein load was visualized in the unstained membrane using BioRad ChemiDoc imager (A). After 1 h blocking with 5% non-fat dry milk, blot 13 was cut above 50 kDa and the bottom part was incubated for LC3 (B). After that the area between 50 and 37 kDa was cut and incubated for β-actin (B). Two stained molecular weights (MW) on each side were used for cutting accuracy. Data from blot 14 were presented in **Figure 2E.**

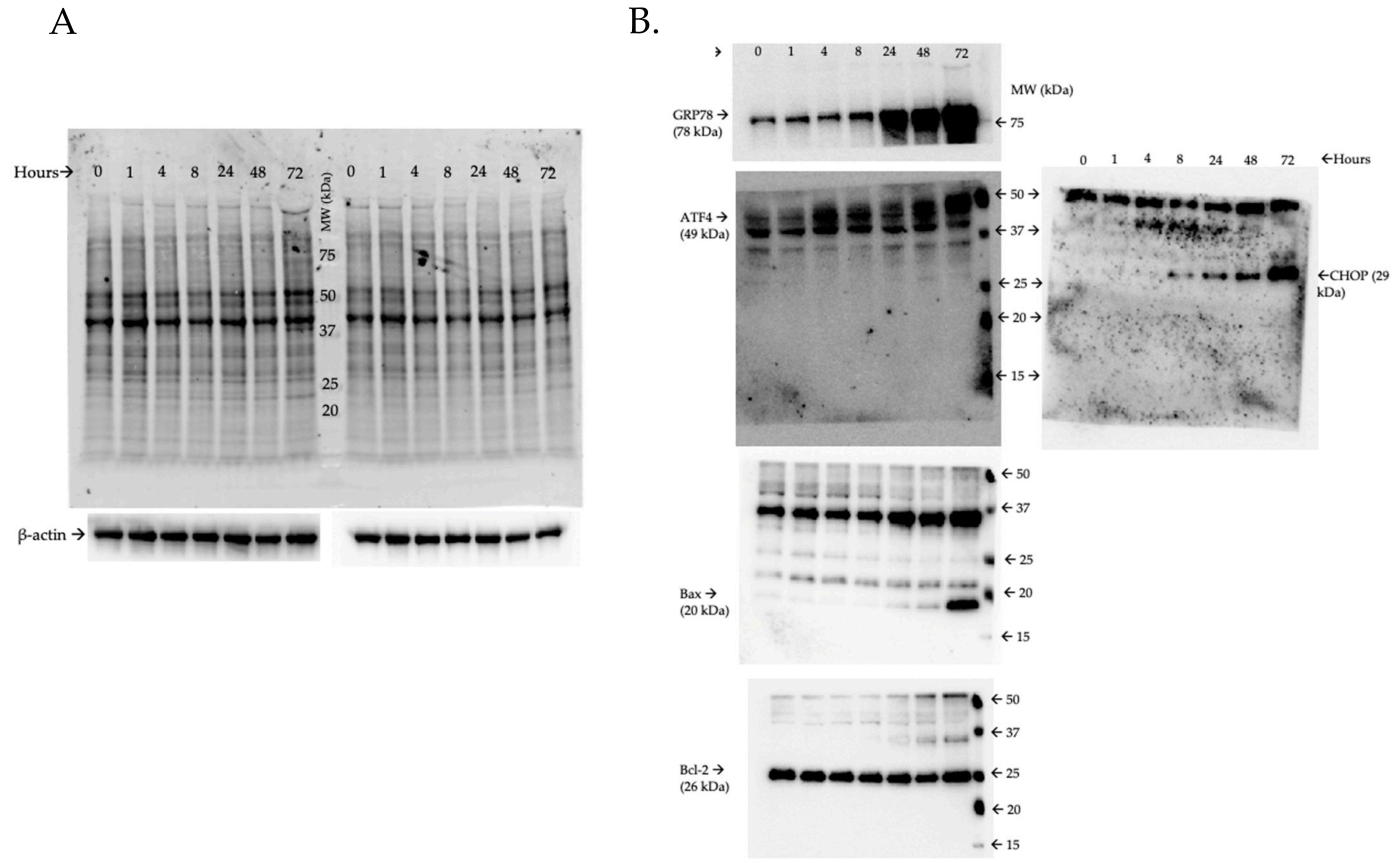

Blot 15. Two identical sets of samples (PEO1 treated with NFV 20 μM at the demonstrated time points were run for 30 min at 200V on TGX stain free fast cast acrylamide gel (12%). After the run, the gel was activated by UV light. Proteins on the gel were transferred using TransBlot Turbo for 7 min. After transfer, total protein load was visualized in the unstained membrane using BioRad Chemi-Doc imager (A). After 1 h of blocking with 5% non-fat dry milk, blot 14 was cut in the middle along the molecular weight ladder to yield two identical sets of blots. The blots were further cut at between 75 kDa and 50 kDa. The top left blot was incubated for GRP78 (B). The bottom two blots were incubated for ATF4 and CHOP respectively (B). Afterwards, the left bottom blot was incubated for Bax and Bcl-2 seqencially. Finally, the blots were incubated for β-actin (A). Multiple stained molecular weights (MW) were used for cutting accuracy. Data from blot 15 were presented in **Figure 3A.**

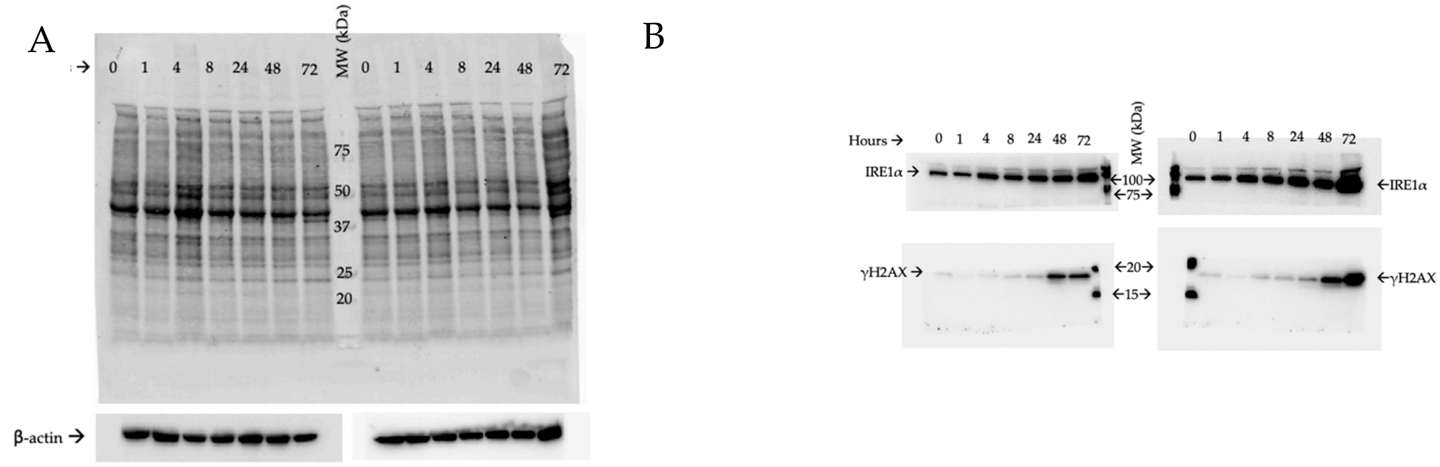

Blot 16. Two identical sets of samples (PEO1 treated with NFV 20 μM on the demonstrated time points were run 30 min at 200V on TGX stain free fast cast acrylamide gel (12%). After the run, the gel was activated by UV light. Proteins on the gel were transferred using TransBlot Turbo for 7 min. After transfer total protein load was visualized in the unstained membrane using BioRad Chemi-Doc imager (A). After 1 h of blocking with 5% non-fat dry milk, blot 15 was cut in the middle along the molecular weight ladder to yield two identical sets of blots. Both blots were again cut below the mark of 75 kDa and above the mark of 20 kDa. The top blots were incubated for IRE1α and the bottom blots were incubated for γH2AX. Finally, the blots were incubated for β-actin. Data from blot 16 were presented in **Figure 3A and 5B.**

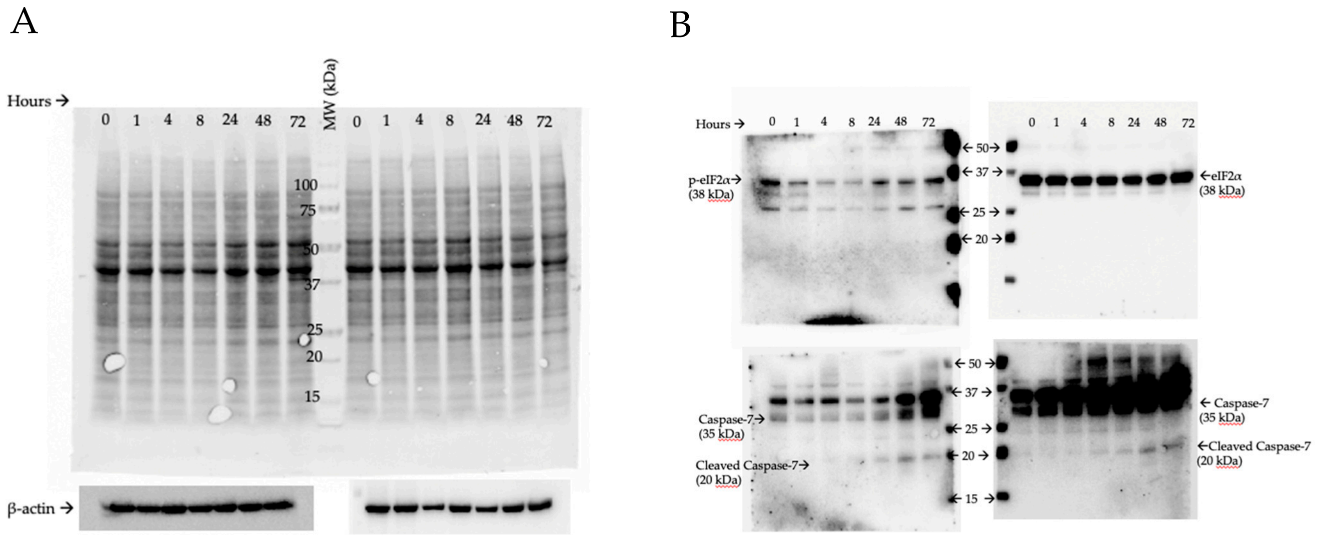

Blot 17. Two identical sets of samples (PEO1 treated with NFV 20 μM on the demonstrated time points were run 30 min at 200V on TGX stain free fast cast acrylamide gel (12%). After the run, the gel was activated by UV light. Proteins on the gel were transferred using TransBlot Turbo for 7 min. After transfer total protein load was visualized in the unstained membrane using BioRad Chemi-Doc imager (A). After 1 h of blocking with 5% non-fat dry milk, blot 11 was cut in the middle along the molecular weight ladder to yield two identical sets of blots. Both blots were again cut below the mark of 75 kDa. The bottom blots were incubated for p-eIF2α (left), eIF2α (right) and casapase-7. Finally, the blots were incubated for β-actin. Data from blot 17 were presented in **Figure 3A and 4D.**

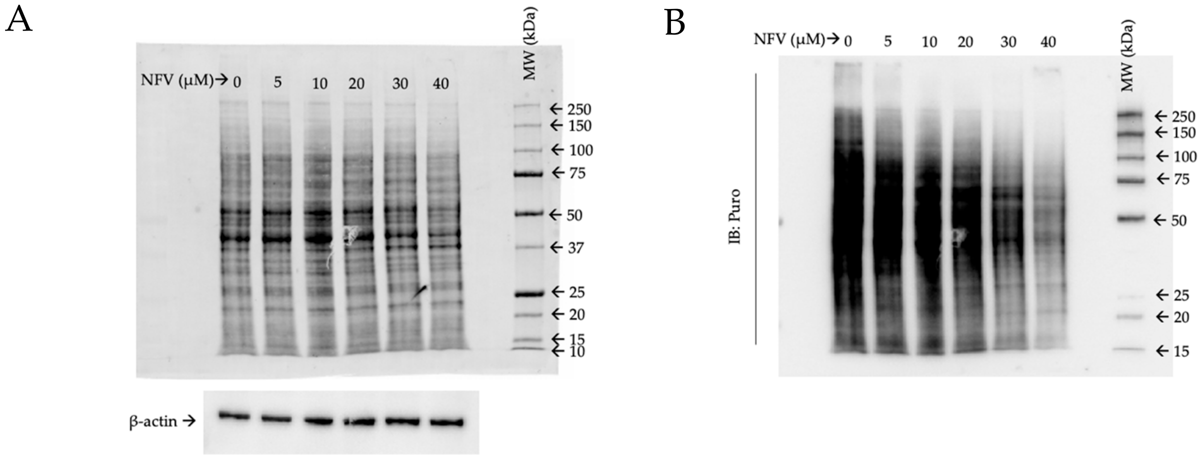

Blot 18. PEO1 cells were treated with 5-40 μM of NFV for 72 h. 30 min before terminating the experiment, the cells were incubated with puromycin at 37∘C to a final concentration of 1 μM. Extracted proteins from the samples were run for 30 min at 200V on TGX stain free fast cast acrylamide gel (10%). After the run, the gel was activated by UV light. Proteins on the gel were transferred using TransBlot Turbo for 7 min. After transfer, total protein load was visualized in the unstained membrane using BioRad ChemiDoc imager (A). After 1 h blocking with 5% non-fat dry milk, the blot was incubated with anti-puromycin antibody (B). Finally, the blot was incubated for β-actin (A). Data from blot 18 were presented in **Figure 4A.**

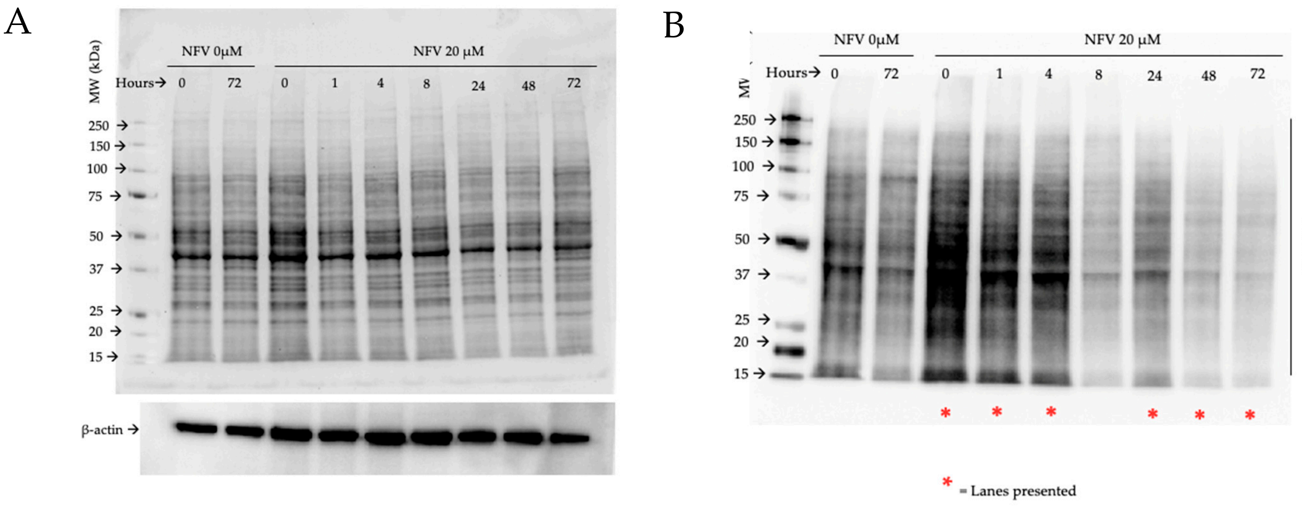

Blot 19. PEO1 cells were treated with or without 20 µM of NFV for the indicated time points in the blot (0-72 h). 30 min before terminating the experiment, the cells were incubated with puromycin at 37◦C to a final concentration of 1 µM. Extracted proteins from the samples were run for 30 min at 200V on TGX stain free fast cast acrylamide gel (10%). After the run, the gel was activated by UV light. Proteins on the gel were transferred using TransBlot Turbo for 7 min. After transfer total protein load was visualized in the unstained membrane using BioRad ChemiDoc imager (A). After 1 h of blocking with 5% non-fat dry milk, the blot was incubated with anti-puromycin antibody (B). Finally, the blot was incubated for β-actin (A). Data from blot 19 were presented in **Figure 4B.**

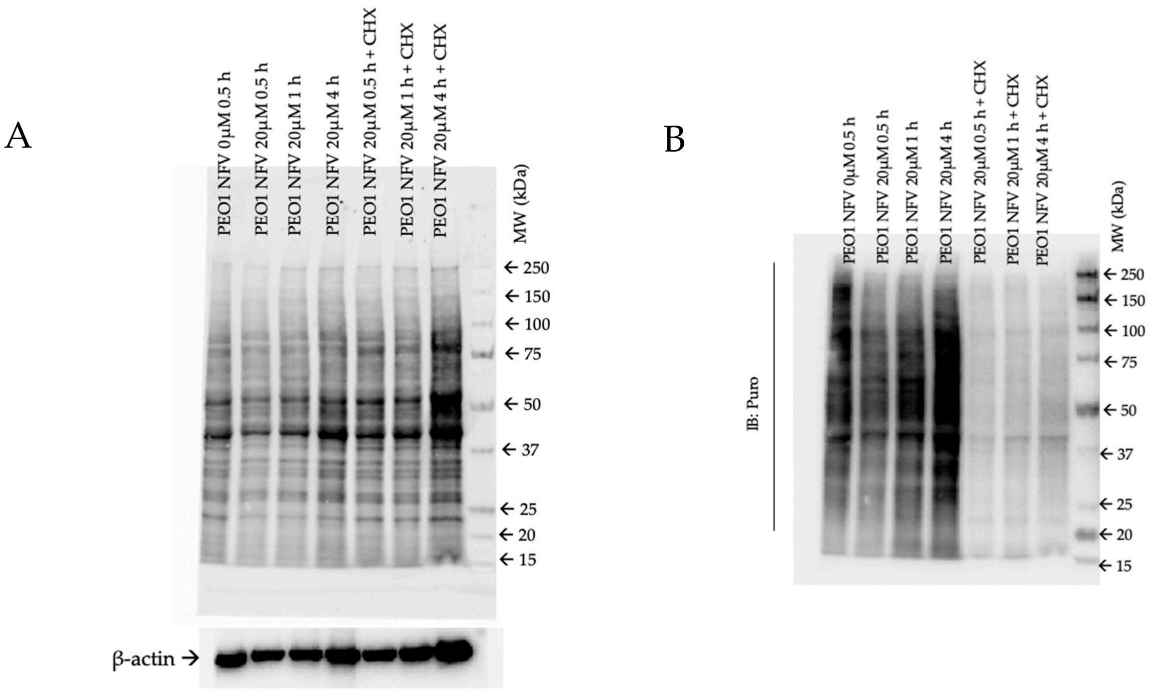

Blot 20. PEO1 cells were treated with 20 µM NFV with or without 10 µg/ml cycloheximide (CHX) for 0.5, 1, or 4 h. 30 min before terminating the experiment, the cells were incubated with puromycin at 37◦C to a final concentration of 1 µM. Extracted proteins from the samples were run for 30 min at 200V on TGX stain free fast cast acrylamide gel (10%). After the run, the gel was activated by UV light. Proteins on the gel were transferred using TransBlot Turbo for 7 min. After transfer, total protein load was visualized in the unstained membrane using BioRad ChemiDoc imager (A). After 1 h of blocking with 5% non-fat dry milk, the blot was incubated with anti-puromycin antibody. Finally, the blot was incubated for β-actin (A). Data from blot 20 were presented in **Figure 4C.**

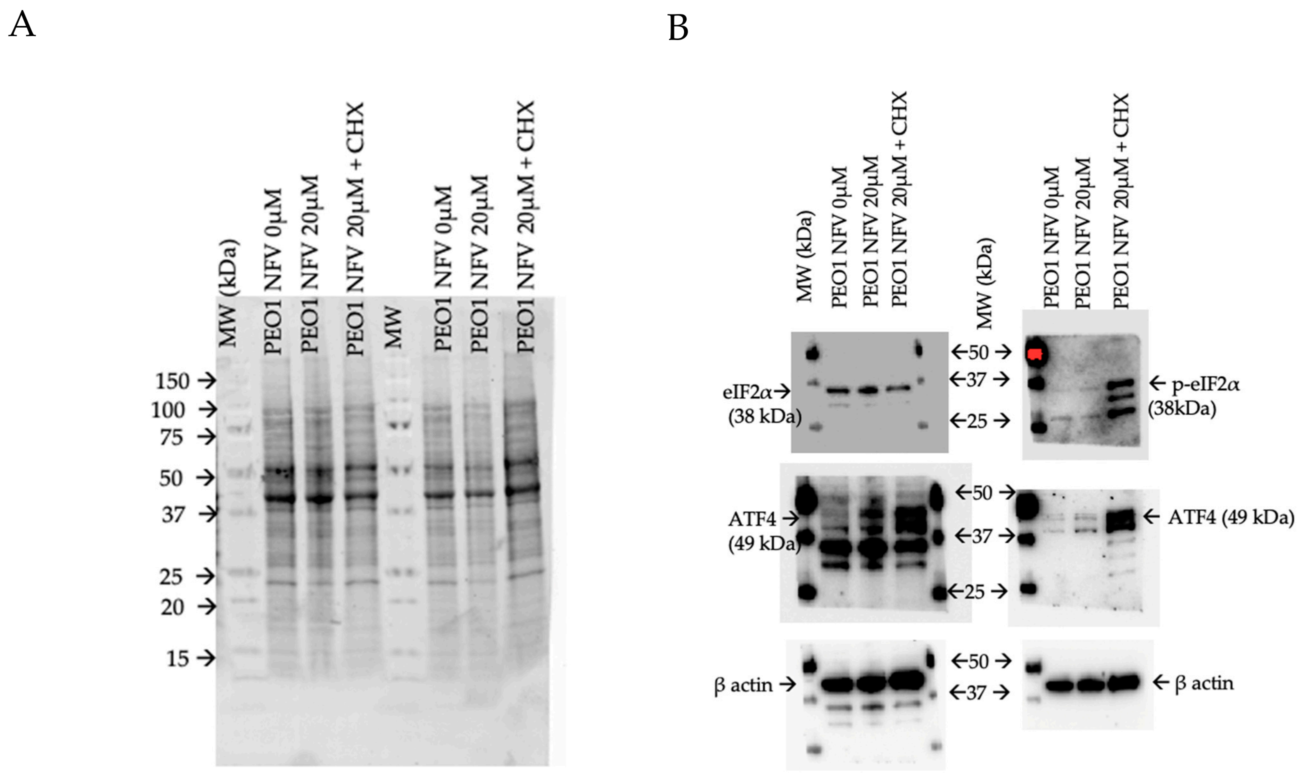

Blot 21. Two identical sets of samples (PEO1 treated with NFV 20 μM with or without cycloheximide (10 μg/mL) for 4 h were run 30 min at 200V on TGX stain free fast cast acrylamide gel (12%). After the run, the gel was activated by UV light. Proteins on the gel were transferred using TransBlot Turbo for 7 min. After transfer, total protein load was visualized in the unstained membrane using BioRad ChemiDoc imager (A). After 1 h of blocking with 5% non-fat dry milk, the blot was cut in the middle along the molecular weight ladder to yield two identical sets of blots. The blots were cut above 50 kDa and below 25 kDa, and the area in between was incubated for p-eIF2α, eIF2α, ATF4, and β-actin (B). Data from blot 21 were presented in **Figure 4E.**

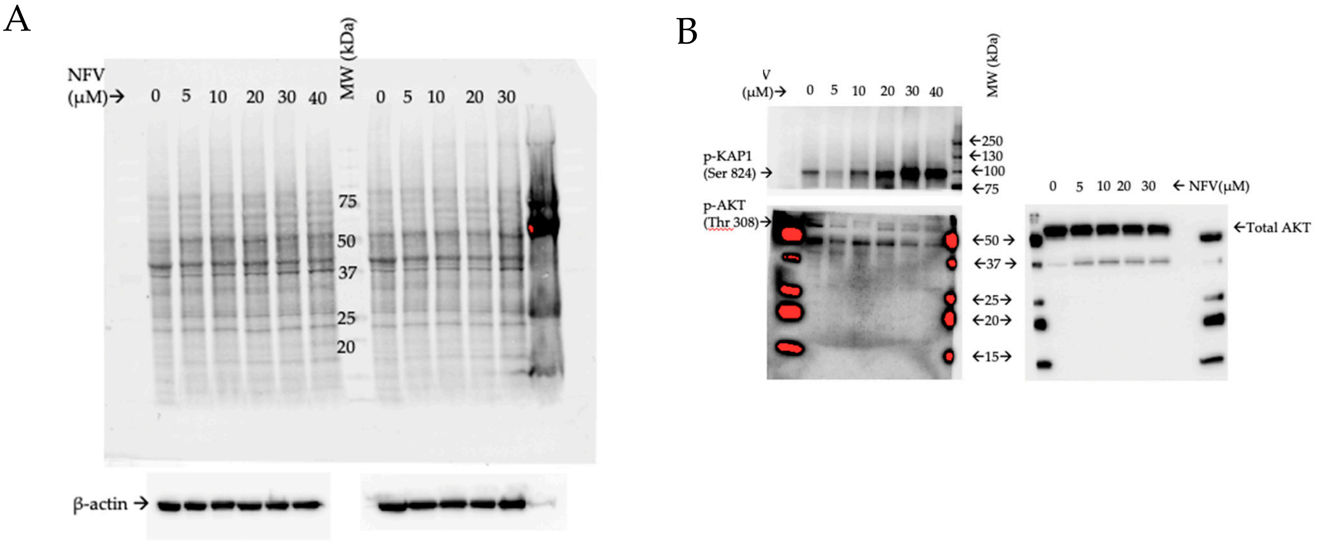

Blot 22. Proteins extracted from PEO1 cells treated with 5-40 μM NFV were run for 30 mi at 200V on TGX stain free fast cast acrylamide gel (12%) in two identical sets. After the run, the gel was activated by UV light. Proteins on the gel were transferred using TransBlot Turbo for 7 min. After transfer, total protein load was visualized in the unstained membrane using BioRad ChemiDoc imager (A). After 1 h of blocking with 5% non-fat dry milk, the blot was cut in the middle along the molecular weight ladder to yield two identical sets of blots. The blots were cut below 75 kDa and below 25 kDa. The top left part was incubated for p-KAP1 (Ser 824) and the bottom parts were incubated for p-AKT (Thr 308)/ total AKT (B). Finally, the blots were incubated for β-actin (A). Multiple stained molecular weights (MW) were used for cutting accuracy. Data from blot 22 were presented in **Figure 5A.**

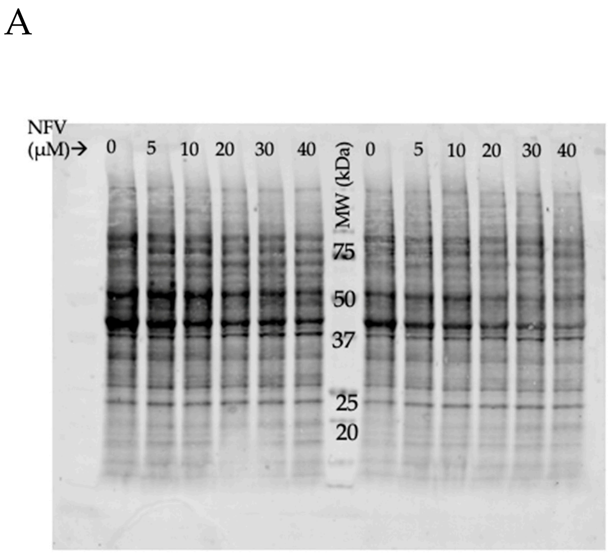

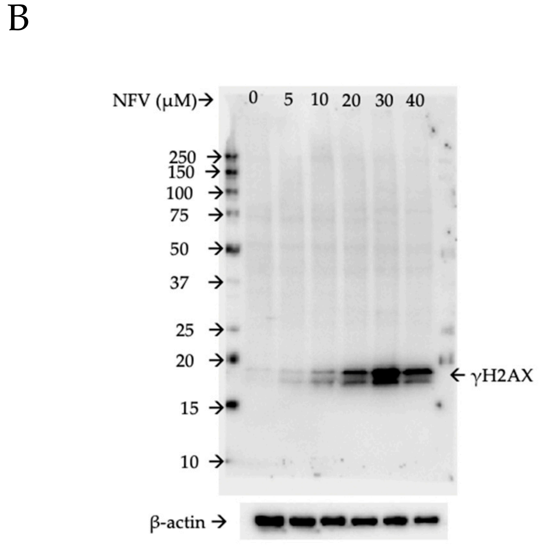

Blot 23. Proteins extracted from PEO1 cells treated with 5-40 μM NFV were run for 30 min at 200V on TGX stain free fast cast acrylamide gel (12%). After the run, the gel was activated by UV light. Proteins on the gel were transferred using TransBlot Turbo for 7 min. After transfer, total protein load was visualized in the unstained membrane using BioRad ChemiDoc imager (A). After 1 h of blocking with 5% non-fat dry milk the membrane was cut in the middle along the molecular weight marker to yield two identical sets of blots, and the right blot was incubated for γH2AX and β-actin sequentially (B). Data from blot 23 were presented in **Figure 5A.**

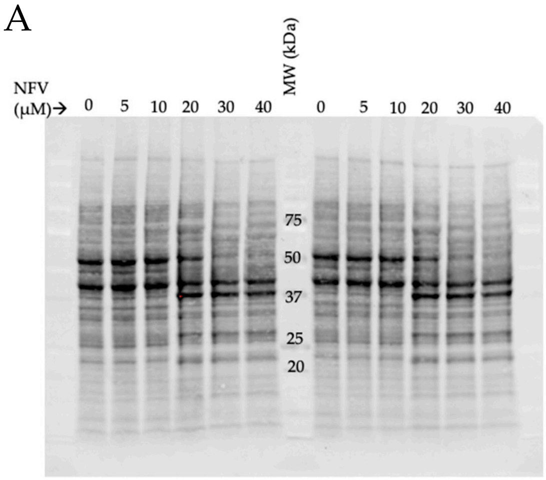

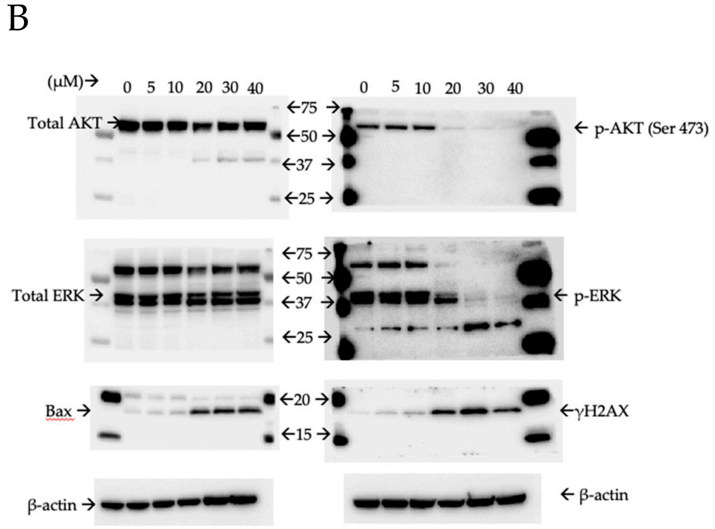

Blot 24. Proteins extracted from PEO4 cells treated with 5-40 μM NFV were run for 30 min at 200V on TGX stain free fast cast acrylamide gel (12%) in two identical sets. After the run, the gel was activated by UV light. Proteins on the gel were transferred using TransBlot Turbo for 7 min. After transfer, total protein load was visualized in the unstained membrane using BioRad ChemiDoc imager (A). After 1 h of blocking with 5% non-fat dry milk, the blot was cut in the middle along the molecular weight ladder to yield two identical sets of blots. The blots were cut at 75 kDa and below 25 kDa. The middle parts were incubated for p-AKT (Ser 473)/total AKT, p-ERK/total ERK and β-actin (B). The bottom left blot was incubated for Bax and the bottom right blot was incubated for γH2AX (B). Data from blot 24 were presented in **Figure 3B and 5A.**

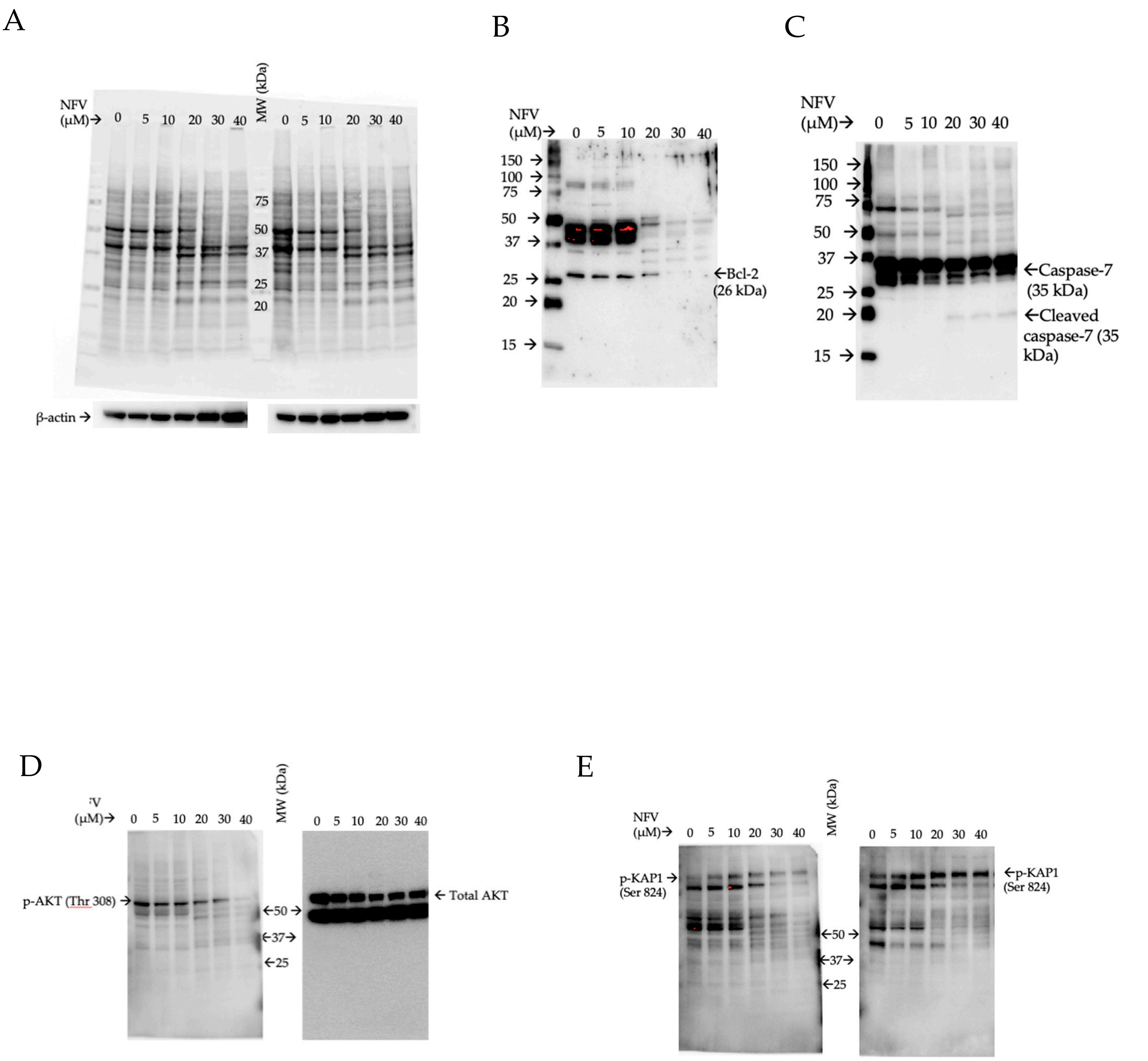

Blot 25. Proteins extracted from PEO4 cells treated with 5-40 μM NFV were run for 30 min at 200V on TGX stain free fast cast acrylamide gel (12%) in two identical sets. After the run, the gel was activated by UV light. Proteins on the gel were transferred using TransBlot Turbo for 7 min. After transfer total protein load was visualized in the unstained membrane using BioRad ChemiDoc imager. After 1 h blocking with 5% non-fat dry milk, the blot was cut in the middle along the molecular weight ladder to yield two identical sets of blots. The left blot was incubated for Bcl-2 (B), p-AKT (Thr 308) (D) and p-KAP1 (Ser 824) (E), and the right blot was incubated for caspase-7 (C), Total AKT (D) and p-KAP1 (Ser 824) (E), sequentially. Finally, both blots were incubated for β-actin (A). Data from blot 25 were presented in **Figure 3B and 5A.**

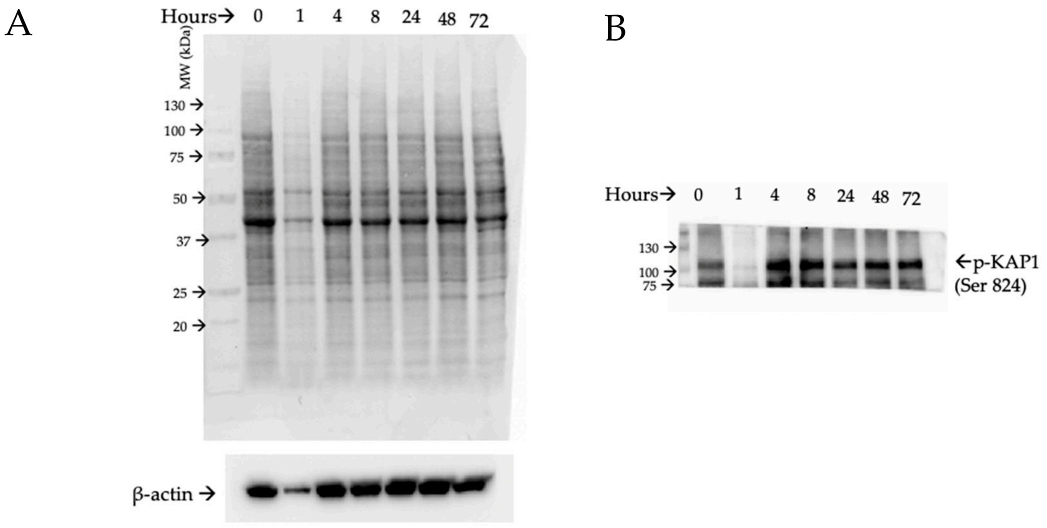

Blot 26. PEO1 treated with NFV 20 μM at the demonstrated time points were run for 30 min at 200V on TGX stain free fast cast acrylamide gel (12%). After the run, the gel was activated by UV light. Proteins on the gel were transferred using TransBlot Turbo for 7 min. After transfer, total protein load was visualized in the unstained membrane using BioRad ChemiDoc imager (A). The blot was cut below 75 kDA and the top part was incubated for p-KAP1 (Ser 824) and the bottom part was incubated for β-actin. Data from blot 26 were presented in **Figure 5B.**

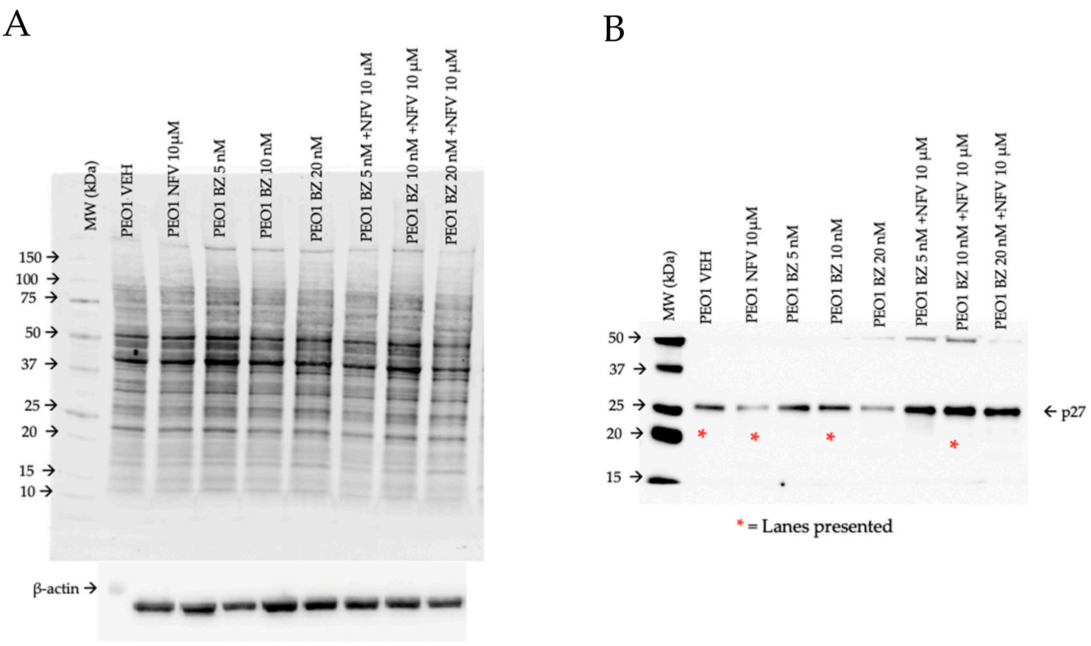

Blot 27. PEO1 cells were treated with 5, 10, or 20 nM bortezomib (BZ) with or without 10 μM NFV for 72 h. Extracted proteins from the samples were run for 30 min at 200V on TGX stain free fast cast acrylamide gel (12%). After the run, the gel was activated by UV light. Proteins on the gel were transferred using TransBlot Turbo for 7 min. After transfer total protein load was visualized in the unstained membrane using BioRad ChemiDoc imager (A). After 1 h of blocking with 5% non-fat dry milk, the blot was cut below 75 kDa and the bottom part was incubated for $p27^{kip1}$ (B). After that the area between 50 and 37 kDa was cut and incubated for β-actin (A). Data from blot 27 were presented in **Figure 6C**

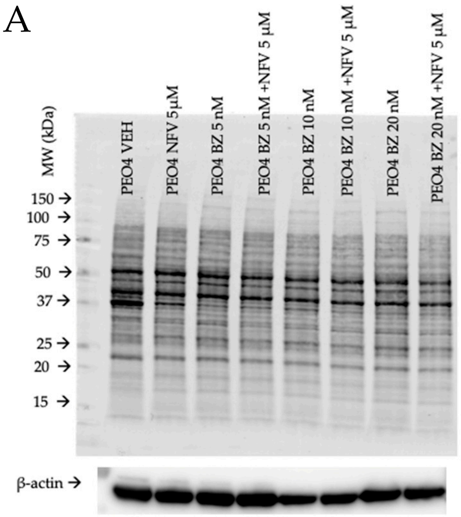

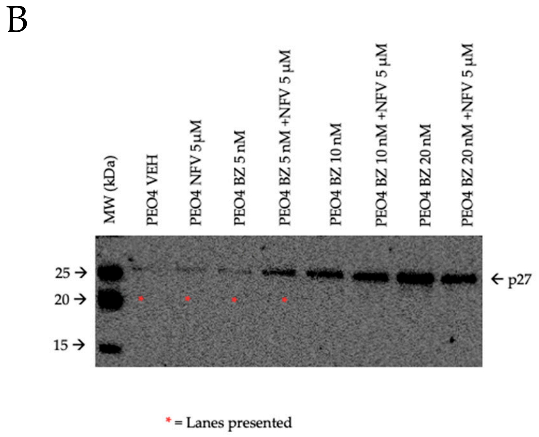

Blot 28. PEO4 cells were treated with 5, 10, or 20 nM bortezomib (BZ) with or without 5 μM NFV for 72 h. Extracted proteins from the samples were run for 30 min at 200V on TGX stain free fast cast acrylamide gel (12%). After the run, the gel was activated by UV light. Proteins on the gel were transferred using TransBlot Turbo for 7 min. After transfer total protein load was visualized in the unstained membrane using BioRad ChemiDoc imager (A). After 1 h blocking with 5% non-fat dry milk, blot was cut below 75 kDa and 37 Kda. The part below 37 kDa was incubated for p27^{kip1} (B) and the part between 50 and 37 kDa was incubated for β-actin (A). Data from blot 28 were presented in **Figure 6G.**

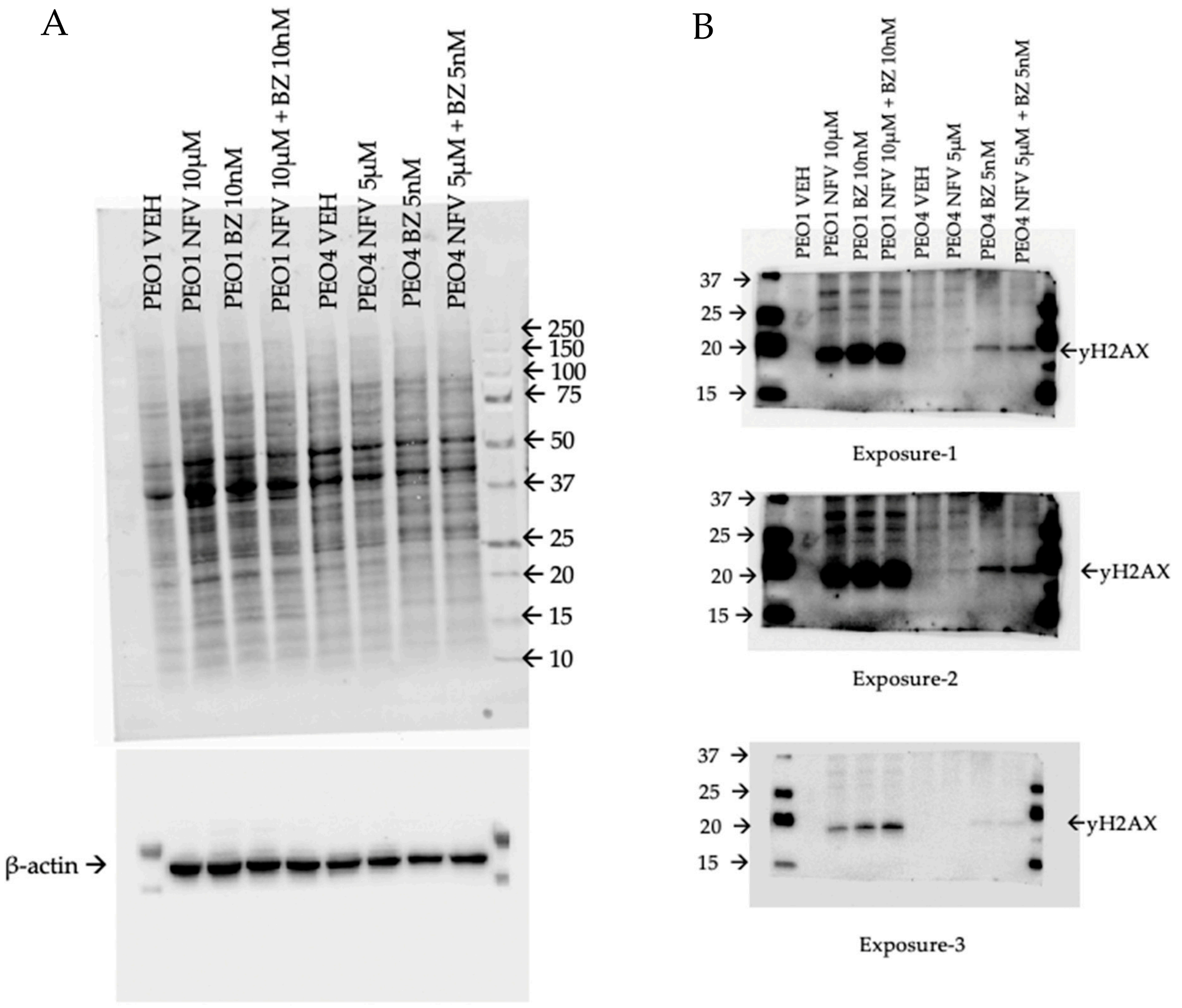

Blot 29. PEO1 cells were treated with 10 nM bortezomib (BZ) with or without 10 μM NFV, and PEO4 cells were treated with 5 nM bortezomib (BZ) with or without 5 μM NFV for 72 h. Extracted proteins from the samples were run for 30 min at 200V on TGX stain free fast cast acrylamide gel (12%). After the run, the gel was activated by UV light. Proteins on the gel were transferred using TransBlot Turbo for 7 min. After transfer total protein load was visualized in the unstained membrane using BioRad ChemiDoc imager (A). After 1 h blocking with 5% non-fat dry milk, blot was cut at 50 kDa and incubated for β-actin (A). Afterwards, the blot was cut at 37 kDa, and was incubated for phosphorylated H2AX (B). Multiple stained molecular weights (MW) were used for cutting accuracy. Data from blot 29 were presented in **Figure 6C and 6G.**

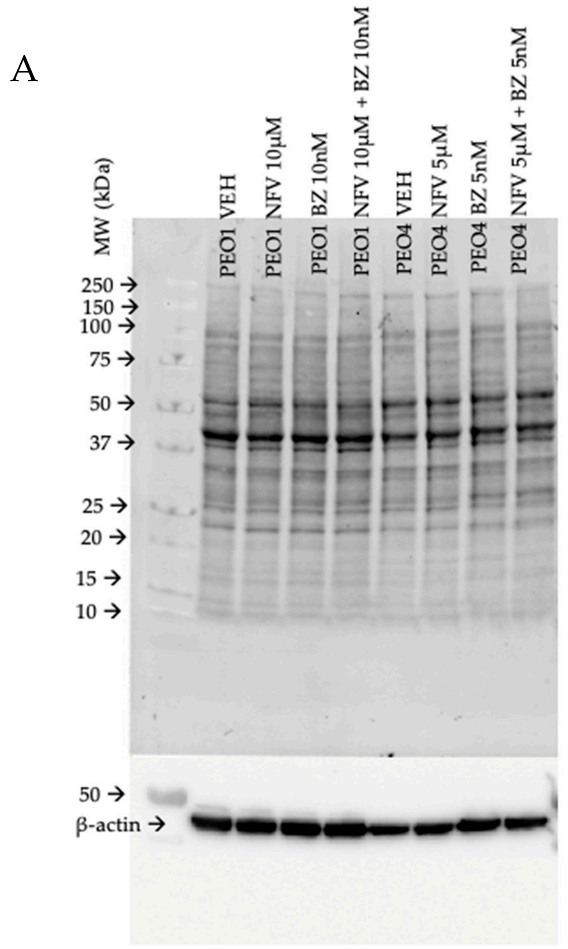

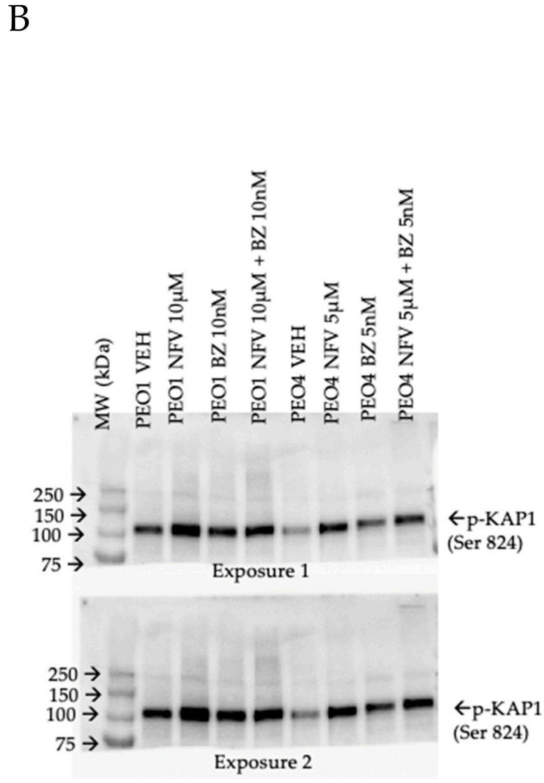

Blot 30. PEO1 cells were treated with 10 nM bortezomib (BZ) with or without 10 μM NFV, and PEO4 cells were treated with 5 nM bortezomib (BZ) with or without 5 μM NFV for 72 hours. Extracted proteins from the samples were run for 30 min at 200V on TGX stain free fast cast acrylamide gel (12%). After the run, the gel was activated by UV light. Proteins on the gel were transferred using TransBlot Turbo for 7 min. After transfer, total protein load was visualized in the unstained membrane using BioRad ChemiDoc imager (A). After 1 h blocking with 5% non-fat dry milk, blot was cut below 75 kDa and the top part was incubated for p-KAP1 (Ser 824) (B) and the bottom part was incubated for β-actin (A). Data from blot 30 were presented in **Figure 6C and 6G.**

A

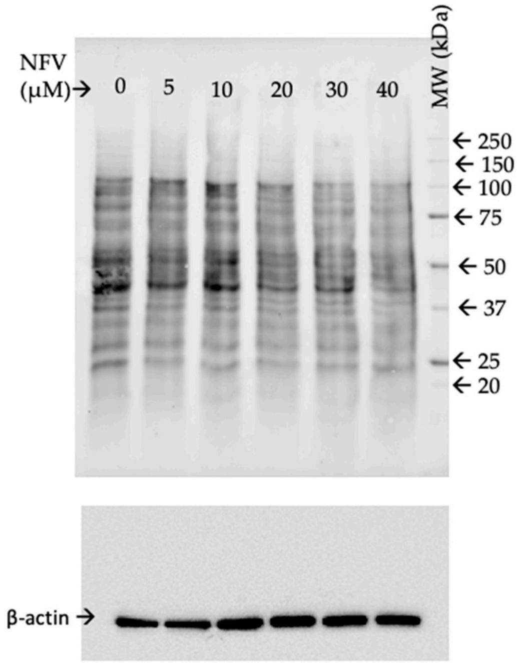

B

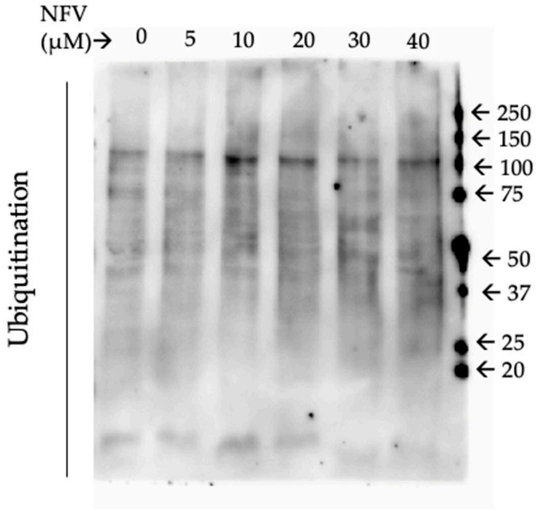

Blot 31. Proteins extracted from PEO1 cells treated with 5-40 μM NFV were run for 30 min at 200V on TGX stain free fast cast acrylamide gel (10%). After the run, the gel was activated by UV light. Proteins on the gel were transferred using TransBlot Turbo for 7 min. After transfer total protein load was visualized in the unstained membrane using BioRad ChemiDoc imager (A). After 1 h of blocking with 5% non-fat dry milk, the blot was incubated for ubiquitin (B) and β-actin (A). Data from blot 31 were presented in **Figure 6I.**

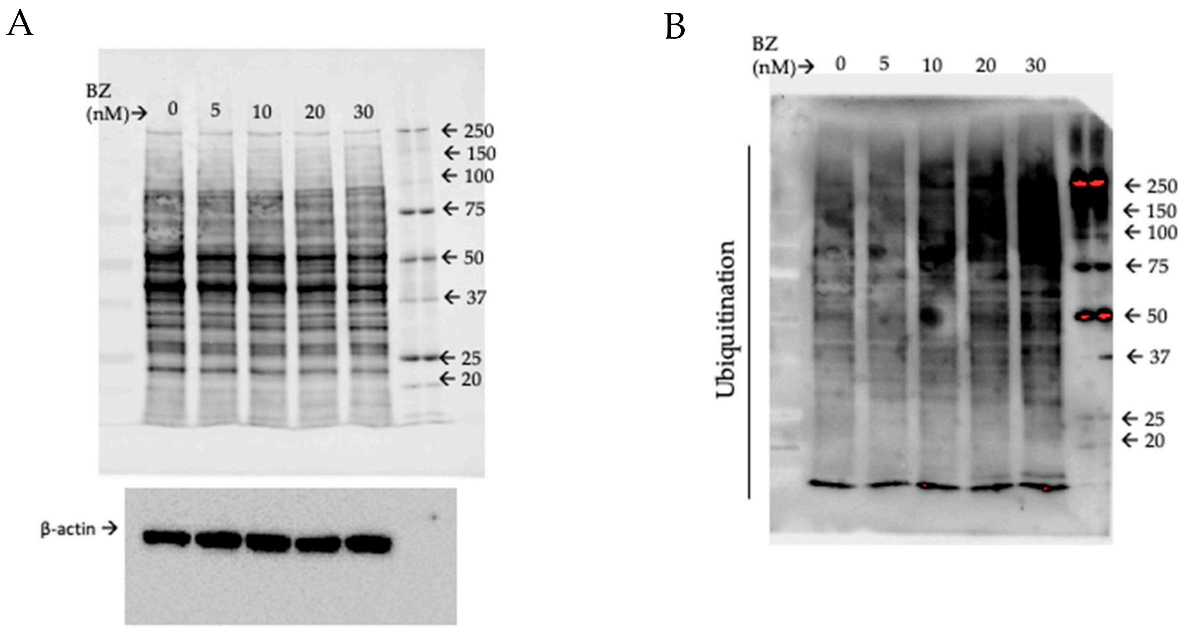

Blot 32. Proteins extracted from PEO1 cells treated with 5-40 nM BZ were run for 30 minutes at 200V on TGX stain free fast cast acrylamide gel (10%). After the run, the gel was activated by UV light. Proteins on the gel were transferred using TransBlot Turbo for 7 min. After transfer total protein load was visualized in the unstained membrane using BioRad ChemiDoc imager (A). After 1 h of blocking with 5% non-fat dry milk, the blot was incubated for ubiquitin (B) and β-actin (A). Data from blot 32 were presented in **Figure 6J.**

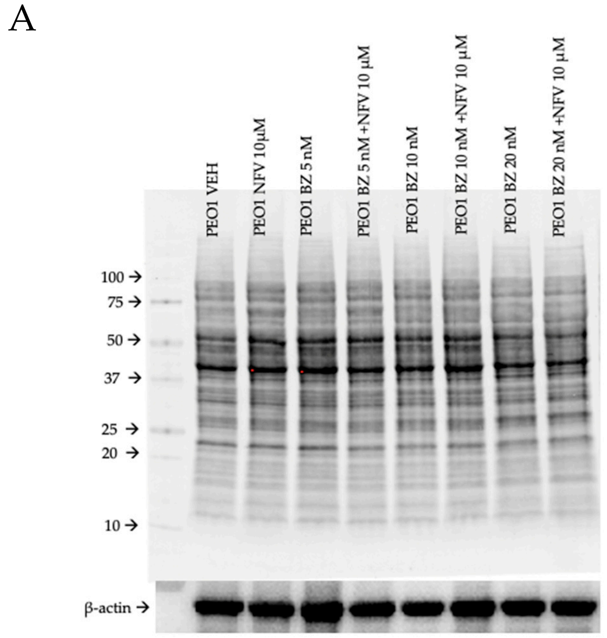

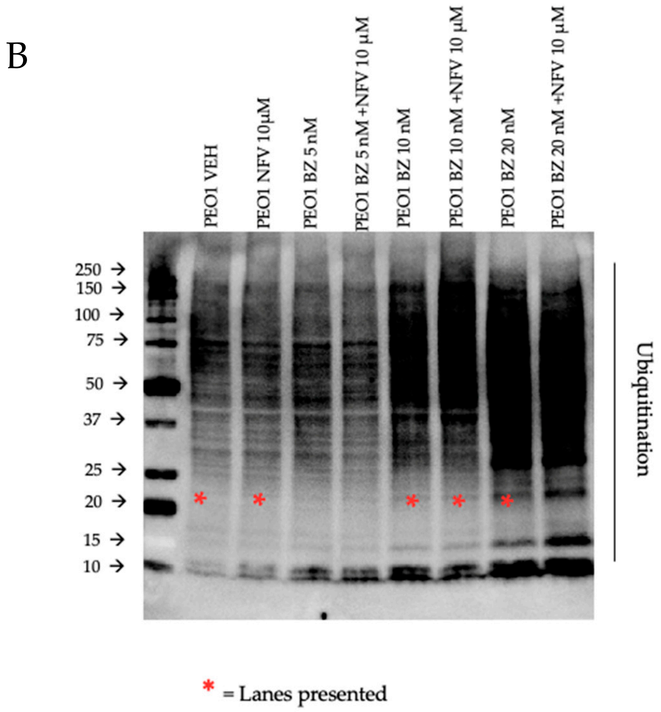

Blot 33. Proteins extracted from PEO1 cells treated with 5, 10, or 20 nM BZ with or without 10 μM NFV for 72 h, were run for 30 min at 200V on TGX stain free fast cast acrylamide gel (10%). After the run, the gel was activated by UV light. Proteins on the gel were transferred using TransBlot Turbo for 7 min. After transfer, total protein load was visualized in the unstained membrane using BioRad ChemiDoc imager (A). After 1 h of blocking with 5% non-fat dry milk, the blot was incubated for ubiquitin (B) and β-actin (A). Data from blot 33 were presented in **Figure 6K.**

Figure S4. Raw western blot data corresponding to the figures shown in the main section of the manuscript.
